# Nanotechnology: The Future Medicine

**DOI:** 10.4103/0974-2077.63301

**Published:** 2010

**Authors:** Rajiv Saini, Santosh Saini, Sugandha Sharma

**Affiliations:** *Department of Periodontology and Oral Implantology, Rural Dental College - Loni, Maharashtra, India*; 1 *Department of Microbiology, Oral Implantology, Rural Dental College - Loni, Maharashtra, India*; 2 *Department of Prosthodontics, Oral Implantology, Rural Dental College - Loni, Maharashtra, India*

**Keywords:** Future, medicine, nanotechnology

## Abstract

Nanotechnology is an exciting new area in science, with many possible applications in medicine. This article seeks to outline the role of different areas such as diagnosis of diseases, drug delivery, imaging, and so on.

## INTRODUCTION

Nanotechnology can be defined as the science and engineering involved in the design, synthesis, characterization, and application of materials and devices whose smallest functional organization, in at least one dimension, is on the nanometer scale or one billionth of a meter. At these scales, consideration of individual molecules and interacting groups of molecules in relation to the bulk macroscopic properties of the material or device becomes important, as it has a control over the fundamental molecular structure, which allows control over the macroscopic chemical and physical properties.[1] Nanotechnology has found many applications in medicine and this articles outlines some such applications.

## POSSIBLE MECHANISMS OF NANOTECHNOLOGY IN RELATION TO MEDICINE

These materials and devices can be designed to interact with cells and tissues at a molecular (i.e., subcellular) level, for applications in medicine and physiology, with a high degree of functional specificity, thus allowing a degree of integration between technology and biological systems not previously attainable. It should be appreciated that nanotechnology is not in itself a single emerging scientific discipline, but rather, a meeting of different traditional sciences, such as, chemistry, physics, materials science and biology, to bring together the required collective expertise needed to develop these novel technologies.[1] The promise that nanotechnology brings is multifaceted, offering not only improvements to the current techniques, but also providing entirely new tools and capabilities.

By manipulating drugs and other materials at the nanometer scale, the fundamental properties and bioactivity of the materials can be altered. These tools can permit a control over the different characteristics of drugs or agents such as:[2]

- alteration in solubility and blood pool retention time
- controlled release over short or long durations
- environmentally triggered controlled release or highly specific site-targeted delivery

## APPLICATIONS OF NANOMATERIALS IN MEDICINE

These applications include fluorescent biological labels, drug and gene delivery, bio-detection of pathogens, detection of protein, probing of DNA structure, tissue engineering, tumor detection, separation and purification of biological molecules and cells, MRI contrast enhancement and phagokinetic studies.[3] The long-term goal of nanomedicine research is to characterize the quantitative molecular-scale components known as nanomachinery. Precise control and manipulation of nanomachinery in cells can lead to better understanding of the cellular mechanisms in living cells, and to the development of advanced technologies, for the early diagnosis and treatment of various diseases. The significance of this research lies in the development of a platform technology that will influence nanoscale imaging approaches designed to probe molecular mechanisms in living cells.[4] Molecular imaging has emerged as a powerful tool to visualize molecular events of an underlying disease, sometimes prior to its downstream manifestation. The merging of nanotechnology with molecular imaging provides a versatile platform for the novel design of nanoprobes that will have tremendous potential to enhance the sensitivity, specificity and signalling capabilities of various biomarkers in human diseases.[5]

Nanoparticle probes can endow imaging techniques with enhanced signal sensitivity, better spatial resolution and the ability to relay information on biological systems at molecular and cellular levels. Simple magnetic nanoparticles can function as magnetic resonance imaging (MRI) contrast enhancement probes. These magnetic nanoparticles can then serve as a core platform for the addition of other functional moieties including fluorescence tags, radionuclides and other biomolecules, for multimodal imaging, gene delivery and cellular trafficking. An (MRI) with hybrid probes of magnetic nanoparticles and adenovirus can detect target cells and monitor gene delivery and expression of green fluorescent proteins optically.[6] Nuclear techniques such as positron-emission tomography (PET) potentially provide detection sensitivities of higher magnitude, enabling the use of nanoparticles at lower concentrations than permitted by routine MRI. Furthermore, a combination of the high sensitivity of PET with the anatomical detail provided by computed tomography (CT) in hybrid imaging, has the potential to map signals to atherosclerotic vascular territories.[7] Molecular imaging always requires accumulation of the contrast agent in the target site, and this can be achieved more efficiently by steering nanoparticles containing the contrast agent into the target. This entails accessing target molecules hidden behind tissue barriers, necessitating the use of targeting groups. For imaging modalities with low sensitivity, nanoparticles bearing multiple contrast groups provide signal amplification. The same nanoparticles can, in principle, deliver both the contrast medium and the drug, allowing monitoring of the bio-distribution and therapeutic activity simultaneously (referred to as theranostics).[8] Such nanofiber-based scaffolds are available in a wide range of pore size distribution, high porosity and high surface area-to-volume ratio. Such a wide range of parameters are favourable for cell attachment, growth and proliferation, and also provide a basis for the future optimization of an electrospun nanofibrous scaffold in a tissue-engineering application.

## CONCLUSIONS

Thus, it is concluded that, nanotechnology or systems / device manufacture at the molecular level, is a multidisciplinary scientific field undergoing explosive development. The genesis of nanotechnology can be traced to the promise of revolutionary advances across medicine, communications, genomics and robotics.
